# Early evaluation of patient risk for substantial weight gain during olanzapine treatment for schizophrenia, schizophreniform, or schizoaffective disorder

**DOI:** 10.1186/1471-244X-8-78

**Published:** 2008-09-15

**Authors:** Ilya Lipkovich, Jennie G Jacobson, Thomas A Hardy, Vicki Poole Hoffmann

**Affiliations:** 1 Eli Lilly and Company; Lilly Corporate Center, Indianapolis, IN 46285 USA

## Abstract

**Background:**

To make well informed treatment decisions for their patients, clinicians need credible information about potential risk for substantial weight gain. We therefore conducted a post-hoc analysis of clinical trial data, examining early weight gain as a predictor of later substantial weight gain.

**Methods:**

Data from 669 (Study 1) and 102 (Study 2) olanzapine-treated patients diagnosed with schizophrenia, schizophreniform, or schizoaffective disorder were analyzed to identify and validate weight gain cut-offs at Weeks 1–4 that were predictive of substantial weight gain (defined as an increase of ≥ 5, 7, 10 kg or 7% of baseline weight) after approximately 30 weeks of treatment. Baseline characteristics alone, baseline characteristics plus weight change from baseline to Weeks 1, 2, 3 or 4, and weight change from baseline to Weeks 1, 2, 3, or 4 alone were evaluated as predictors of substantial weight gain. Similar analyses were performed to determine BMI increase cut-offs at Weeks 1–4 of treatment that were predictive of substantial increase in BMI (1, 2 or 3 kg/m^2 ^increase from baseline).

**Results:**

At Weeks 1 and 2, predictions based on early weight gain plus baseline characteristics were more robust than those based on early weight gain alone. However, by Weeks 3 and 4, there was little difference between the operating characteristics associated with these two sets of predictors. The positive predictive values ranged from 30.1% to 73.5%, while the negative predictive values ranged from 58.1% to 89.0%. Predictions based on early BMI increase plus baseline characteristics were not uniformly more robust at any time compared to those based on early BMI increase alone. The positive predictive values ranged from 38.3% to 83.5%, while negative predictive values ranged from 42.1% to 84.7%. For analyses of both early weight gain and early BMI increase, results for the validation dataset were similar to those observed in the primary dataset.

**Conclusion:**

Results from these analyses can be used by clinicians to evaluate risk of substantial weight gain or BMI increase for individual patients. For instance, negative predictive values based on data from these studies suggest approximately 88% of patients who gain less than 2 kg by Week 3 will gain less than 10 kg after 26–34 weeks of olanzapine treatment. Analysis of changes in BMI suggests that approximately 84% of patients who gain less than .64 kg/m^2 ^in BMI by Week 3 will gain less than 3 kg/m^2 ^in BMI after 26–34 weeks of olanzapine treatment. Further research in larger patient populations for longer periods is necessary to confirm these results.

## Background

Obesity and diabetes represent a growing problem in the general population of the United States [1,2]. These disorders are present at even greater rates in individuals with schizophrenia [3,4]. Many factors contribute [5], including lack of exercise [6], lack of adequate medical care [7], and a possible predisposition toward weight gain in general [8] or in association with improvement of symptoms [9,10]. Weight gain associated with antipsychotic treatment for schizophrenia has been commonly reported [11,12]. Clinicians are increasingly aware of the need to balance the potential risks of weight gain and other adverse events against the benefits of treatment for control of symptoms and improvements in quality of life. To make well-informed treatment decisions for their patients, clinicians need credible information about patient risk for substantial weight gain.

It has recently been shown that lack of response to antipsychotic treatment at 2 weeks is a good predictor of poor longer-term response to treatment [13,14]. In addition, weight gain of at least 2.0 kg at 3 weeks after initiation of olanzapine is a robust predictor of substantial weight gain (defined as gaining at least 5 kg or 7% of baseline body weight) at 30 weeks in individuals with bipolar disorder [15] and weight gain of at least 7% of baseline body weight during the first 6 weeks of olanzapine treatment for schizophrenia has been shown to be associated with greater weight gain after 1 year of treatment [16]. To determine whether weight gain within the first few weeks of olanzapine treatment for schizophrenia can predict later substantial weight gain, we conducted post-hoc analyses of data from 2 randomized, controlled clinical trials of olanzapine treatment in this patient population.

These analyses used 4 different threshold values (increase of 5, 7, 10 kg, and 7% from baseline weight) in defining substantial weight gain at endpoint so that clinicians can choose the threshold most relevant for a particular patient. Linear discriminant analysis was used to derive early weight gain thresholds for predicting substantial weight gain. Similar analyses were also performed using early change in body mass index (BMI) to predict later substantial increase in BMI. Weight and BMI data from Weeks 1, 2, 3, and 4 were used because these time points may be relevant for clinicians who have initiated patients on olanzapine for reasons of acute efficacy and are making risk/benefit-based decisions about continuing or changing treatment.

## Methods

### Data source

Post-hoc analyses were conducted using data from a large, randomized, controlled clinical trial within the Eli Lilly and Company database, and repeated using a smaller trial from the same database to test whether results were reproducible. In both studies, patients were diagnosed with schizophrenia, schizophreniform, or schizoaffective disorders; and weight was assessed weekly for the first 4 weeks of treatment and again at 28–30 weeks. For both studies, all patients provided informed written or witnessed oral consent after the study was described to them.

Study 1 was a 52-week trial comparing olanzapine (5–20 mg/day) and haloperidol (5–20 mg/day), conducted from June 1993 to February 1995 [17]. Of the 1336 patients who were randomized to treatment with olanzapine, 669 remained in the study and had weight recorded at approximately Week 30 (occurring between 26 and 34 weeks after initiation of study drug due to variation in visit intervals allowed by the protocol); of these, 661 had height data available allowing for calculation of BMI. The analysis population study included patients who were initially randomized to and continuously treated with olanzapine, up to 30 +/- 4 weeks, either within the double-blind arm or after being switched to open-label olanzapine rescue.

Study 2 was a 28-week, randomized, double-blind trial comparing olanzapine (10–20 mg/day) and risperidone (4–12 mg/day), conducted from April 1995 to January 1997 [18]. Of the 172 olanzapine-treated patients, 102 had a recorded weight at endpoint; of these, 101 had height data available, allowing for calculation of BMI.

### Variables

These analyses examined 4 different definitions of substantial weight gain at endpoint (5, 7, 10 kg, and 7% of baseline weight) as well as 3 different definitions of substantial BMI increase (1, 2 and 3 kg/m^2^). Two different endpoint times (Week 30 for Study 1 and Week 28 for Study 2) were used due to the differences in scheduled visit intervals. These time points were chosen in order have comparable endpoints for both studies and because they are within the 21- to 39-week time period when weight gain associated with olanzapine treatment has been reported to plateau [19,20].

Early weight gain at Weeks 1, 2, 3, and 4 was evaluated for usefulness in predicting risk of substantial weight gain. Early increase in BMI at Weeks 1, 2, 3, and 4 were evaluated for usefulness in predicting risk of substantial increase in BMI. Baseline variables examined as potential predictors of weight gain were age, Caucasian race, sex, smoking status and body mass index (BMI; kg/m^2^) [21].

### Statistical methodology

The majority of analyses were performed for both weight gain (using early weight gain to predict substantial weight gain at endpoint) and BMI increase (using early BMI increase to predict substantial increase in BMI at endpoint). Analyses are explained below using weight gain as an example, but analogous analyses were also performed examining increase in BMI. Prediction rules for substantial weight gain were constructed using linear discriminant analysis (LDA, implemented using SAS^® ^PROC DISCRIM) for several sets of predictors: (i) baseline characteristics alone, (ii) baseline characteristics and weight change from baseline at specific early time points (Weeks 1, 2, 3, and 4), (iii) early weight change as a single predictor. In LDA, a prediction rule is based on classification score (or linear discriminant function) calculated for discriminating between groups (for instance patients with and patients without substantial weight gain). The classification score is based on the sum of scores for each individual predictor evaluated for every patient by multiplying each predictor's value by its associated coefficient in the linear discriminant function. This total score is compared with a threshold value to differentiate between patients predicted to and predicted not to experience substantial weight gain. The coefficients for individual predictors are estimated from the data so as to achieve the best discrimination between the two groups (in terms of the ratio of between-to-within group variation) using all predictors in the model. The threshold is estimated to ensure that patients are allocated to the group for which they have the largest probability of membership. When using early weight change as a single predictor, the threshold simplifies to the midpoint between the average early weight change in groups with and without substantial weight gain.

In using predictive models with multiple predictors, the classification score is based on a combination of the amount of early weight change and other variables included in the model. As a result, there is no single cut-off point for early weight gain that can be used to predict whether or not a patient will experience substantial weight gain. The threshold applies to the entire linear combination of multiple predictors, which implies that for the same amount of early weight gain, patients may be predicted to gain substantial weight or not gain substantial weight depending on the values for other characteristics included in the prediction model.

The performance of a classification rule can be evaluated by examining its operating characteristics (sensitivity, specificity, and positive and negative predictive values). To calculate these, the analysis population is divided into four groups:

∘ Group A–patients for whom the rule correctly predicted substantial weight gain;

∘ Group B–patients for whom the rule incorrectly predicted substantial weight gain (false positive);

∘ Group C–patients for whom the rule incorrectly predicted no substantial weight gain (false negatives); and

∘ Group D–patients for whom the rule correctly predicted no substantial weight gain.

The operating characteristics of a classification rule can then be calculated:

∘ *Sensitivity *measures how well the classification rule predicts which patients would gain substantial weight. It is the percentage of all patients with substantial weight gain (Groups A and C combined) who were predicted to have substantial weight gain (Group A).

∘ *Specificity *indicates how well the classification rule predicts which patients will not have substantial weight gain. It is the percentage of all patients without substantial weight gain (Groups B and D combined) who were predicted not to gain substantial weight (Group D).

∘ *Positive predictive value *indicates how likely a prediction of substantial weight gain is to be correct. It is the percentage of all patients predicted to have substantial weight gain (Groups A and B combined) for whom that prediction was correct (Group A).

∘ *Negative predictive value *indicates how likely a prediction of no substantial weight gain is to be correct. It is the percentage of all patients predicted not to gain substantial weight (Groups C and D combined) for whom that prediction was correct (Group D).

Because operating characteristics of a classification rule computed using the same data that were used to derive the rule (data re-substitution) are inherently over-optimistic, we used the method of leave-one-out cross-validation to obtain more realistic estimates of operating characteristics. In this method, each patient was predicted to have substantial weight gain or to have no substantial weight gain based on a prediction rule formed by using only the data from the remaining (*n*-1) patients. The method of cross-validation typically results in a smaller number of correct predictions, compared with data re-substitution, which better reflects performance of a prediction rule expected with new data. Also cross-validation allows for a fairer comparison of operating characteristics across prediction rules involving different numbers of predictors, appropriately penalizing for "overfitting" the data when including too many predictors in the model. Finally, we applied the prediction rules derived from the primary dataset to an independent validation dataset (Study 2) and determined the resulting operating characteristics.

To evaluate the direction in which patient drop out may have affected characteristics of our analysis population (subjects available at week 30) we constructed logistic regression models for probability that a patient was included in the analysis population using as predictors patient's characteristics available at baseline and changes in psychiatric symptoms and weight at Week 3.

To examine how patient discontinuation could have affected the early weight gain cut-offs reported here, we performed a sensitivity analysis using multiple imputation [22,23], imputing missing values for 30-week weight data for Study 1 patients who were available up to at least Week 3. Each missing value was imputed 100 times resulting in 100 completed datasets. The imputation was based on Bayesian regression that imputes missing observations for every patient using all available data (i.e., all repeated measures on weight up to the patient's discontinuation from the study). The imputation takes advantage of correlation in repeated measures; however, it also explicitly incorporates uncertainty in missing values about the expected trends (i.e. it does not simply impute future weight as values predicted from a linear regression on the previous values). As a result, multiple completed datasets were generated, mimicking the variability expected in missing observations. This procedure automatically adjusted for bias in observed data induced by the selection mechanism to the extent that the variables driving the selection bias were utilized in the imputation procedure. Imputation obviously cannot make up for any bias explained by variables that were not available or not included in the imputation model. Using imputation, we completed 100 datasets; each contained data on N = 1156 patients (number of olanzapine-treated patients available at Week 3). We evaluated the cut-offs for early weight gain and associated operating characteristics for each of these 100 datasets; summaries over 100 datasets (means, max and min values of operating characteristics) were reported.

## Results

### Baseline characteristics and weight change in Studies 1 and 2

Detailed demographics and other information on Studies 1 and 2 have already been published [16-18,21]. Briefly, olanzapine-treated patients in both studies were predominantly male, predominantly Caucasian with similar mean ages, and mean baseline BMIs (Table 1). In Study 1, lower BMI, lower age, male sex, non-Caucasian race and weight gain at Weeks 1 through 4 were all significantly correlated with later weight increase. In addition, lower BMI, younger age and weight gain at Weeks 1 through 4 were significantly correlated with gaining at least 7% of baseline weight. Endpoint for Study 1 in these analyses was 30 weeks, with a protocol allowed variation of 4 weeks. In Study 2, which had a smaller patient population, lower BMI and weight gain at Weeks 2 through 4 were significantly correlated both with weight change at endpoint and with gaining at least 7% of baseline weight by Week 28 (Table 1). Discontinuation due to weight gain as an adverse event was rare in both studies, occurring in less than 1% of olanzapine-treated patients.

**Table 1:** Predictors of Significant Weight Gain: Patient Characteristics and Weight Changes During Acute Olanzapine Treatment

	Study 1 (N = 669)			Study 2 (N = 102)		
	
Predictors of weight gain	Value	Correlation^a ^with weight change at endpoint	Correlation^a ^with 7% WG at endpoint	Value	Correlation^a ^with weight change at endpoint	Correlation^a ^with 7% WG at endpoint
Baseline BMI,^b ^kg/m^2^	26.1 (5.1)	- 0.16***	- 0.20***	26.7 (5.6)	- 0.21*	- 0.25*
Age,^b ^years	38.9 (11.5)	- 0.18***	- 0.18***	35.9 (10.8)	- 0.09	- 0.02
Caucasian^c ^N (%)	549 (82.1%)	- 0.08*	- 0.06	81 (79.4%)	- 0.13	- 0.10
Men^c ^N (%)	431 (64.4%)	0.08*	0.04	68 (66.7%)	- 0.06	- 0.06
Weight change from baseline^b^						
At Week 1, kg	0.50 (1.7)	0.28***	0.24***	0.47 (1.9)	0.19	0.17
At Week 2, kg	1.1 (2.1)	0.41***	0.34***	1.0 (2.3)	0.44***	0.35***
At Week 3, kg	1.5 (2.5)	0.46***	0.39***	1.6 (2.4)	0.43***	0.33***
At Week 4, kg	1.9 (2.6)	0.53***	0.45***	2.0 (2.5)	0.51***	0.44***
At endpoint,^d ^kg	5.4 (6.9)			5.2 (6.6)		

^a ^Pearson coefficient of correlation

^b^Value shown as mean (standard deviation)

^c^Value shown as number (percent)

^d^Week 26 to 34

P-values for correlation with endpoint weight change, *p < .05, ***p < .001

Abbreviations: BMI, body mass index; WG, weight gain

**Figure 1:**
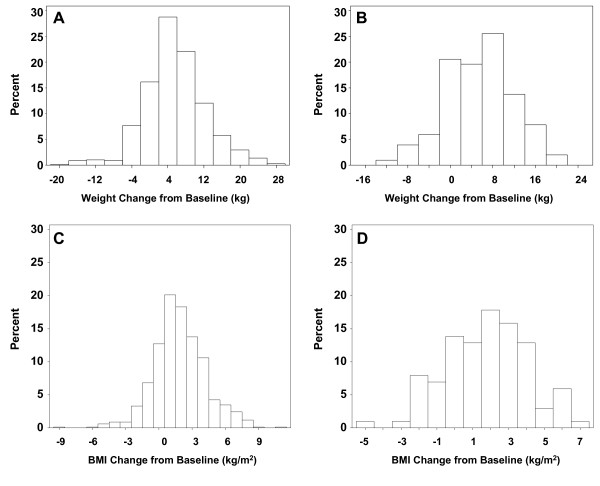
**Weight change (kg) and BMI change (kg/m^2^) from baseline to 30-week endpoint in Studies 1 and 2**. For Study 1 (N = 669), the mean (standard deviation [SD]) weight change was 5.4 kg (6.9); for Study 2 (N = 102), it was 5.2 kg (6.6). Data were grouped into intervals of 4 kg with midpoints of each interval shown along horizontal axes. The height of each bar corresponds to the proportion of patients whose weight change falls within that range. For Study 1 (N = 661), the mean (SD) BMI change was 1.9 (2/4) kg/m^2^; for Study 2, (N = 101), the mean (SD) BMI change was 1.8 (2.3) kg/m^2^. Data were grouped in intervals of 1 kg/m^2 ^with midpoints of each interval shown along the horizontal axes. The height of each bar corresponds to the proportion of patients whose BMI change falls within that range.

In Study 1, change in weight at 30 weeks among olanzapine-treated patients ranged from a decrease of 22 kg to a gain of 27 kg; mean change was a gain of 5.4 kg (Figure 1A). In Study 2, change in weight at 28 weeks ranged from a decrease of 13 kg to a gain of 20 kg; mean change was a gain of 5.2 kg at Week 28 (Figure 1B). Magnitude and variability of weight change was similar between studies.

In Study 1, change in BMI at 30 weeks among olanzapine treated patients ranged from a decrease of 8.5 kg/m^2 ^to an increase of 10.8 kg/m^2^; mean change was an increase of 1.9 kg/m^2 ^(Figure 1C). In Study 2, change in BMI at 28 weeks ranged from a decrease of 5.3 kg/m^2 ^to an increase of 6.8 kg/m^2^, mean change was a gain of 1.8 kg/m^2 ^(Figure 1D).

### Operating characteristics for predicting substantial weight gain

Tables 2 and 3 present operating characteristics for different definitions of substantial weight gain (Column 1) and predictors included in the model (Column 2) based on prediction rules derived from Study 1. Two sets of estimated operating characteristics are reported: the first was derived from Study 1 data (via leave-one-out cross-validation) and the second was obtained by applying the rules derived from Study 1 data to the validation data from Study 2. Also shown are the number and percent of patients who met the conditions of the prediction rules and who experienced the defined level of substantial weight gain or substantial BMI increase at the endpoint. In general, sensitivity and specificity improve as time of early weight/BMI assessment increases, indicating that predictions made based on data from Weeks 3 or 4 have greater discriminating power than those based on data from earlier time points.

**Table 2:** Operating Characteristics for Various Predictive Models and Definitions of Substantial Weight Gain

		Evaluated from Study 1								Based on validation dataset (Study 2)			
		
SWG^a^	Predictive Model^b^	EWG cut-off	N	Sensitivity,^c ^%	Specificity,^d ^%	PPV^e ^%	NPV^f ^%	+TEST n (%)^g^	+SWG n (%)^h^	Sensitivity,^i ^%	Specificity,^j ^%	PPV^k ^%	NPV^l ^%
5 kg	WG at 1 wk	0.5 kg	669	54.0	65.5	61.6	58.1	297 (44.4)	339 (50.7)	49.1	73.9	69.2	54.8
5 kg	WG at 2 wks	1.1 kg	667	60.1	71.1	68.1	63.4	298 (44.7)	338 (50.7)	56.4	70.2	68.9	57.9
5 kg	WG at 3 wks	1.4 kg	669	65.9	70.2	69.6	66.6	322 (48.1)	340 (50.8)	63.6	70.2	71.4	62.3
5 kg	WG at 4 wks	1.9 kg	668	70.9	72.8	72.6	71.1	329 (49.3)	337 (50.4)	67.3	71.7	74.0	64.7
7 kg	WG at 1 wk	0.6 kg	669	54.7	68.3	53.1	69.7	273 (40.8)	265 (39.6)	53.7	76.7	61.1	70.8
7 kg	WG at 2 wks	1.3 kg	667	62.5	69.7	57.5	74.0	287 (43.0)	264 (39.6)	65.9	70.5	60.0	75.4
7 kg	WG at 3 wks	1.7 kg	669	68.4	71.2	61.1	77.4	298 (44.5)	266 (39.8)	73.2	73.8	65.2	80.4
7 kg	WG at 4 wks	2.2 kg	668	69.2	73.6	63.0	78.6	289 (43.3)	263 (39.4)	68.3	80.0	70.0	78.7
7 kg	BC alone	N/A	667	62.4	54.7	47.3	69.1	347 (52.0)	263 (39.4)	70.7	38.3	43.9	65.7
7 kg	WG at 1wk+BC	N/A	661	60.3	62.9	51.6	70.7	306 (46.3)	262 (39.6)	65.9	57.6	51.9	70.8
7 kg	WG at 2wks+BC	N/A	660	66.7	66.9	56.9	75.4	306 (46.4)	261 (39.5)	68.3	65.0	57.1	75.0
7 kg	WG at 3wks+BC	N/A	661	68.4	70.6	60.6	77.2	297 (44.9)	263 (39.8)	68.3	68.3	59.6	75.9
7 kg	WG at 4wks+BC	N/A	660	71.9	72.8	63.2	80.0	296 (44.8)	260 (39.4)	80.5	76.3	70.2	84.9
10 kg	WG at 1 wk	0.7 kg	669	53.0	64.1	30.1	82.4	266 (39.8)	151 (22.6)	45.8	68.8	31.4	80.3
10 kg	WG at 2 wks	1.5 kg	667	55.7	68.9	34.0	84.4	244 (36.6)	149 (22.3)	58.3	67.9	35.9	84.1
10 kg	WG at 3 wks	2.0 kg	669	68.2	68.7	38.9	88.1	265 (39.6)	151 (22.6)	66.7	67.9	39.0	86.9
10 kg	WG at 4 wks	2.6 kg	668	68.5	71.7	41.0	88.8	249 (37.3)	149 (22.3)	66.7	76.6	47.1	88.1
10 kg	BC alone	N/A	667	60.3	55.6	28.4	82.7	320 (48)	151 (22.6)	75.0	39.0	27.7	83.3
10 kg	WG at 1wk+BC	N/A	661	60.9	63.7	33.2	84.6	277 (41.9)	151 (22.8)	54.2	52.6	26.5	78.4
10 kg	WG at 2wks+BC	N/A	660	63.1	67.9	36.4	86.3	258 (39.1)	149 (22.6)	58.3	63.6	33.3	83.1
10 kg	WG at 3wks+BC	N/A	661	70.9	70.0	41.2	89.0	260 (39.3)	151 (22.8)	70.8	68.8	41.5	88.3
10 kg	WG at 4wks+BC	N/A	660	67.1	71.6	40.8	88.2	245 (37.1)	149 (22.6)	79.2	69.7	45.2	91.4
7%	WG at 1 wk	0.7%	669	53.8	66.0	59.9	60.2	292 (43.6)	325 (48.6)	50.9	75.0	69.2	58.1
7%	WG at 2 wks	1.6%	667	61.0	69.5	65.2	65.5	302 (45.3)	323 (48.4)	62.3	71.4	70.2	63.6
7%	WG at 3 wks	2.0%	669	69.2	72.1	70.1	71.3	321 (48.0)	325 (48.6)	64.2	75.5	73.9	66.1
7%	WG at 4 wks	2.7%	668	70.6	76.2	73.5	73.5	310 (46.4)	323 (48.4)	73.6	75.0	76.5	72.0
7%	BC alone	N/A	667	64.2	57.2	58.2	63.3	354 (53.1)	321 (48.1)	66.0	56.3	62.5	60.0
7%	WG at 1wk+BC	N/A	661	60.6	63.9	61.2	63.4	317 (48.0)	320 (48.4)	66.0	68.1	70.0	64.0
7%	WG at 2wks+BC	N/A	660	62.3	66.7	63.5	65.5	312 (47.3)	318 (48.2)	62.3	68.8	68.8	62.3
7%	WG at 3wks+BC	N/A	661	65.3	71.3	68.1	68.6	307 (46.4)	320 (48.4)	64.2	70.8	70.8	64.2
7%	WG at 4wks+BC	N/A	660	69.2	72.8	70.3	71.8	313 (47.4)	318 (48.2)	69.8	80.9	80.4	70.4

^a^SWG (substantial weight gain) defined as at least 5, 7, 10 kg, and 7% increase from baseline at Week 30 (Study 1) or Week 28 (Study 2).

^b^For each combination of SWG definition (≥ 5, 7, 10 kg, or 7%) and set of predictors (baseline characteristics [BC] and/or weight gain [WG] at 1, 2, 3, or 4 weeks), the prediction rules (classification scores) were constructed with linear discriminant analysis models. When early weight gain is used as a single predictor, the estimated cut-off for early weight gain (in kg or % change) is shown at the time at which early weight gain was assessed. Baseline characteristics, when included in the prediction rules for substantial weight gain (alone or in combination with early weight gain) were age, sex, BMI, and Caucasian ethnicity.

^c^Sensitivity, proportion of patients with SWG that were correctly predicted (estimated using leave-one-out cross validation).

^d^Specificity, proportion of patients with no SWG that were correctly predicted (estimated using leave-one-out cross validation).

^e^Positive predictive value, proportion of patients predicted to have SWG who had substantial weight gain (estimated using leave-one-out cross validation).

^f^Negative predictive value, proportion of patients predicted not to have SWG who did not have substantial weight gain (estimated using leave-one-out cross validation).

^g^Number of patients (%) predicted to have substantial weight gain.

^h^Number of patients (%) who met the criteria for substantial weight gain as defined in the first column of this table. Number and percent of patients with SWG for a given defined SWG vary slightly because values are based on subjects included in the model who had non-missing values,

^i^Sensitivity of prediction rule derived from Study 1 data as estimated from validation data (Study2).

^j^Specificity of prediction rule derived from Study 1 data as estimated from validation data (Study2).

^k^Positive predictive value of prediction rule derived from Study 1 as estimated from validation data (Study2).

^l^Negative predictive value of prediction rule derived from Study 1 as estimated from validation data (Study2).

Abbreviations: SWG, substantial weight gain; EWG, early weight gain; PPV, positive predictive value; NPV, negative predictive value; WG, weight gain; N/A, not applicable.

**Table 3:** Operating Characteristics for Various Predictive Models and Definitions of Substantial BMI Increase

		Evaluated from Study 1								Based on validation dataset (Study 2)			
		
SWG^a ^(kg/m^2^)	Predictive Model^b^	BMIC cut-off	N	Sensitivity,^c^%	Specificity,^d ^%	PPV^e ^%	NPV^f ^%	+TEST n (%)^g^	+SWG N (%)^h^	Sensitivity,^i ^%	Specificity,^j ^%	PPV^k ^%	NPV^l ^%
1	BMIC at 1 wk	.13	661	56.8	63.1	73.9	44.3	329 (49.8)	428 (64.8)	53.1	69.4	75.6	45.5
1	BMIC at 2 wks	.30	660	63.8	68.1	78.7	50.5	347 (52.6)	428 (64.9)	57.8	62.2	72.6	46.0
1	BMIC at 3 wks	.40	661	64.5	68.7	79.1	51.3	349 (52.8)	428 (64.8)	60.9	62.2	73.6	47.9
1	BMIC at 4 wks	.53	660	71.0	74.3	83.5	58.3	363 (55.0)	427 (64.7)	68.8	66.7	78.6	54.6
1	BC alone	N/A	667	59.4	53.8	70.1	42.1	365 (54.7)	431 (64.6)	67.2	54.1	71.7	48.8
1	BMIC at 1 wk+BC	N/A	661	61.0	58.4	72.9	44.9	358 (54.2)	428 (64.8)	70.3	69.4	80.4	56.8
1	BMIC at 2wks+BC	N/A	660	63.6	62.5	75.8	48.2	359 (54.4)	428 (64.9)	65.6	64.9	76.4	52.2
1	BMIC at 3wks+BC	N/A	661	67.8	67.8	79.5	53.4	365 (55.2)	428 (64.8)	64.1	73.0	80.4	54.0
1	BMIC at 4wks+BC	N/A	660	69.8	71.2	81.6	56.3	365 (55.3)	427 (64.7)	67.2	66.7	78.2	53.3
2	BMIC at 1 wk	.18	661	53.4	66.1	56.4	63.3	282 (42.7)	298 (45.1)	50.0	76.0	67.6	60.3
2	BMIC at 2 wks	.39	660	59.6	68.0	60.4	67.3	293 (44.4)	297 (45.0)	60.0	70.6	66.7	64.3
2	BMIC at 3 wks	.52	661	67.2	68.0	63.4	71.5	317 (48.0)	299 (45.2)	66.0	70.6	68.8	67.9
2	BMIC at 4 wks	.69	660	69.9	72.8	67.7	74.9	306 (46.4)	296 (44.9)	66.0	74.0	71.7	68.5
2	BC alone	N/A	667	58.2	52.2	49.7	60.6	350 (52.5)	299 (44.8)	72.0	39.2	53.7	58.5
2	BMIC at 1wk+BC	N/A	661	59.1	60.9	55.4	64.4	318 (48.1)	298 (45.1)	62.0	62.0	62.0	62.0
2	BMIC at 2wks+BC	N/A	660	62.3	64.5	58.9	67.6	314 (47.6)	297 (45.0)	66.0	70.6	68.8	67.9
2	BMIC at 3wks+BC	N/A	661	66.6	67.7	63.0	71.0	316 (47.8)	299 (45.2)	64.0	70.6	68.1	66.7
2	BMIC at 4wks+BC	N/A	660	69.9	71.4	66.6	74.5	311 (47.1)	296 (44.9)	72.0	76.0	75.0	73.1
3	BMIC at 1 wk	.24	661	54.6	66.3	38.3	79.3	261 (39.5)	183 (27.7)	44.8	69.0	37.1	75.4
3	BMIC at 2 wks	.48	660	58.2	68.6	41.4	81.2	256 (38.8)	182 (27.6)	62.1	69.4	45.0	82.0
3	BMIC at 3 wks	.64	661	66.9	69.2	45.6	84.4	270 (40.9)	184 (27.8)	72.4	73.6	52.5	86.9
3	BMIC at 4 wks	.83	660	66.5	70.5	46.2	84.7	262 (39.7)	182 (27.6)	69.0	78.9	57.1	86.2
3	BC alone	N/A	667	58.7	54.9	33.1	77.7	326 (48.9)	184 (27.6)	69.0	43.1	32.8	77.5
3	BMIC at 1wk+BC	N/A	661	64.5	65.5	41.7	82.8	283 (42.8)	183 (27.7)	58.6	56.3	35.4	76.9
3	BMIC at 2wks+BC	N/A	660	62.6	67.2	42.1	82.5	271 (41.1)	182 (27.6)	65.5	68.1	45.2	83.1
3	BMIC at 3wks+BC	N/A	661	66.3	69.0	45.2	84.1	270 (40.9)	184 (27.8)	69.0	69.4	47.6	84.8
3	BMIC at 4wks+BC	N/A	660	66.5	68.6	44.7	84.3	271 (41.1)	182 (27.6)	72.4	71.8	51.2	86.4

^a^SWG (substantial weight gain) defined as an increase of at least 1, 2, or 3 kg/m^2 ^in BMI from baseline at Week 30 (Study 1) or Week 28 (Study 2).

^b^For each combination of SWG definition (≥ 1, 2 or 3 kg/m^2^) and set of predictors (baseline characteristics [BC] and/or BMI Change [BMIC] at 1, 2, 3, or 4 weeks), the prediction rules (classification scores) were constructed with linear discriminant analysis models. When early BMI Change is used as a single predictor, the estimated cut-off for early BMI change is shown at the time at which early BMI change was assessed. Baseline characteristics, when included in the prediction rules for substantial BMI increase (alone or in combination with early BMI change) were age, sex, BMI, and Caucasian ethnicity.

^c^Sensitivity, proportion of patients with SWG that were correctly predicted (estimated using leave-one-out cross validation).

^d^Specificity, proportion of patients with no SWG that were correctly predicted (estimated using leave-one-out cross validation).

^e^Positive predictive value, proportion of patients predicted to have SWG who had substantial weight gain (estimated using leave-one-out cross validation).

^f^Negative predictive value, proportion of patients predicted not to have SWG who did not have substantial weight gain (estimated using leave-one-out cross validation).

^g^Number of patients (%) predicted to have substantial weight change.

^h^Number of patients (%) who met the criteria for substantial BMI change as defined in the first column of this table. Number and percent of patients with SWG for a given defined SWG vary slightly because values are based on subjects included in the model who had non-missing values,

^i^Sensitivity of prediction rule derived from Study 1 data as estimated from validation data (Study2).

^j^Specificity of prediction rule derived from Study 1 data as estimated from validation data (Study2).

^k^Positive predictive value of prediction rule derived from Study 1 as estimated from validation data (Study2).

^l^Negative predictive value of prediction rule derived from Study 1 as estimated from validation data (Study2).

Abbreviations: SWG, substantial weight gain; PPV, positive predictive value; NPV, negative predictive value; N/A, not applicable.

**Figure 2:**
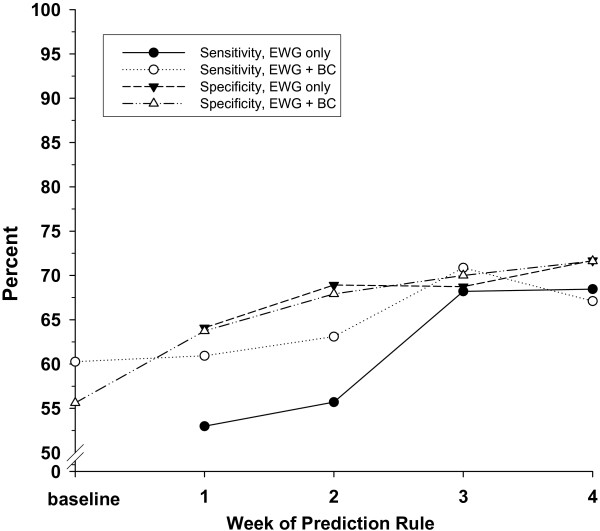
**Specificity and sensitivity for predicting significant weight gain of ≥ 10 kg at 30-week endpoint**. Specificity and sensitivity (%) for predicting significant weight gain of ≥ 10 kg at 30-week endpoint were estimated via leave-one-out cross validation using data from Study 1 and different sets of predictors. The black symbols indicate prediction rules obtained using single cut-offs for early weight change at Weeks 1, 2, 3, and 4 (on horizontal axes). The white symbols indicate prediction rules incorporating baseline characteristics only (shown for Week = 0), and a combination of baseline characteristics and early weight change at Weeks 1, 2, 3, and 4.

Figure 2 shows specificities and sensitivities for Study 1 when substantial weight gain is defined as at least 10 kg at Week 30, allowing comparison of the discriminating ability of predictive models depending on time point for early weight gain and inclusion of baseline characteristics in the predictive model. For all measures, the accuracy improves as time of prediction moves from Week 1 to Week 3 and then appears to level off. In addition, inclusion of baseline characteristics have little or no effect on specificity, suggesting early weight gain without baseline characteristics may be sufficient for predicting which patients will not gain substantial weight. For sensitivity, once early weight gain is included in the prediction model, the additional predictive value of baseline characteristics diminishes and becomes marginal after 3 weeks.

While sensitivity and specificity are important for assessing discriminatory power of prediction rules, positive and negative predictive values (PPV and NPV) may be of more practical interest to clinicians interested in predicting how likely a given patient is to gain substantial amount of weight. Both PPV and NPV tend to increase at later time points, but the greater the defined level of substantial weight gain or BMI increase, the greater the NPV and the smaller the PPV. This is not surprising, as it is easier to predict a more common event (gain of ≥ 5 kg) than a less common one (gain of ≥ 10 kg).

**Figure 3:**
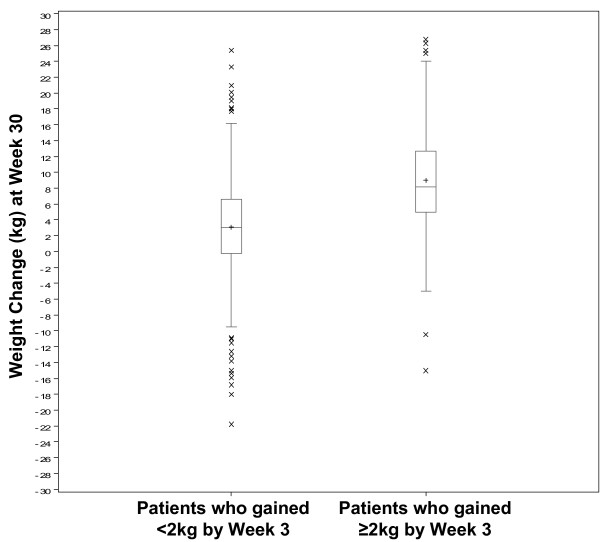
**Distribution of weight change at 30-week endpoint by cut-off of early weight gain**. This figure shows the distribution of weight change at endpoint for patients who did or did not gain at least 2 kg within 3 weeks of treatment. Box plots show the median, and upper and lower quartiles of the distribution as the middle and outer lines of the box. The mean value for each plot is indicated by a +. The upper (lower) fences indicate upper (lower) quartile plus (minus) 1.5 of interquartile range. Observations falling outside fences are shown as X's.

As would be expected from the prediction rule when substantial weight gain is defined as a gain or 10 kg or more, based on a cut-off of weight gain at 3 weeks (Table 2), weight change distribution at endpoint for patients in Study 1 who gained less than 2 kg by Week 3 differs substantially from that of patients who gained 2 kg or more (Figure 3). Weight loss during treatment was much more common among the first group, and weight gain of as much as 25 kg appears to be more common among the second group.

### Effect of smoking status

Smoking status was not available in Study 2. In Study 1, smoking status appeared to have a moderate correlation with short-term weight change, as smokers had less weight gain during the first few weeks of treatment after adjusting for their baseline demographics (at Week 3, *r *= -0.07, p = .088). However, smoking status at baseline had no apparent impact on weight change at later endpoints. Not surprisingly, adding baseline smoking status to the list of predictors of weight gain did not result in improvement of operating characteristics (results not shown). Analyzing data from smokers only (N = 382 at Week 30), the cut-offs for predicting weight gain of 10 kg or more at Week 3 was estimated at 1.9 kg; for gain of 7 kg or more, the cut-off was 1.6 kg, and for gain of 5 kg or more, the cut-off was 1.4 kg (compared with 2.0 kg, 1.7 kg, and 1.4 kg for the overall group, respectively).

### Evaluating potential impact of dropouts on the results

Of necessity, patients who discontinued early from these studies were not included in the analyses. To evaluate the existence and direction of any selection bias resulting from this, variables available at baseline and Week 3 were evaluated as predictors of the probability that patients were included in the analysis population. Caucasian race, age, sex, and baseline BMI did not predict inclusion in the analysis population. Change in Positive and Negative Syndrome Scale (PANSS) total score at Week 3 was highly predictive of remaining in the study at Week 30: (OR = 0.985; CI 0.978–0.992, p < .0001). This is expected since lack of efficacy is a common reason for treatment discontinuation. Weight change at Week 3 was a significant predictor positively associated with probability of remaining in the study by Week 30 (OR = 1.067, CI 1.019–1.118, p = .0063), which is not surprising since weight change was associated with improvement in PANSS total score (e.g. at Week 3, *r *= 0.127, p < .0001). However, when both change in PANSS total score and weight change at Week 3 were included in the same logistic regression model, the adjusted odds ratio for weight was still significant at 5% level. For PANSS total change: OR = 0.986; CI 0.979–0.993 (p < .0001) and for weight change: OR = 1.056, CI 1.007–1.108 (p = 0.0251), indicating that improvement in schizophrenia symptomology and weight gain may have both contributed to the likelihood of patients remaining in this clinical trial.

These results suggest that the weight gain observed in our analysis population at Week 30 may be somewhat larger than the gain that would have been observed if all patients available at 3 weeks had remained in the study up to Week 30. However, from this observation alone it is not clear whether selection bias in weight change for our analysis population could have impacted our estimated cut-offs for early weight gain as predictors of substantial weight gain, and associated operating characteristics (sensitivity, specificity, PPV, NPV).

To evaluate whether patient discontinuations could have biased the results, we performed a sensitivity analysis using multiple imputation [22,23], imputing 30-week weight data for patients who were available up to at least Week 3 and generating 100 completed datasets. Reported below are summary characteristics computed across these 100 datasets that reflect the hypothetical results that would be expected had no dropouts occurred; the difference between maximum and minimum values for the completed datasets reflect uncertainty associated with missing data. Mean change in weight from baseline to endpoint was 4.5 kg (minimum: 4.1 kg, maximum: 5.1 kg), somewhat lower than in the observed data. Correlations between weight change at Week 3 and weight change at endpoint across completed datasets, mean *r *= 0.50 (minimum: 0.43, maximum: 0.56), were somewhat higher than correlations in the observed data (*r *= 0.46) suggesting that dropouts could have caused some dilution of the relationship between early and late weight change in the observed data. Cut-off values for early weight gain used in predicting substantial weight gain at 30 weeks were similar to those obtained with the observed data. For example, for predicting weight gain of 10 kg, mean cut-off for early weight gain at Week 3 was 1.9 kg (minimum: 1.8 kg, maximum: 2.1 kg). For weight gain of 7% the mean cut-off for the percent weight change at Week 3 was 1.9% (minimum: 1.8%, maximum: 2.0%). Operating characteristics for weight gain of 10 kg with early weight gain at Week 3 were: mean sensitivity = 66.1% (minimum: 61.8%, maximum: 70.1%), mean specificity = 70.1% (minimum: 67.5%, maximum: 75.4%), mean PPV = 37.5% (minimum: 32.8%, maximum: 43.1%), and mean NPV = 88.4% (minimum: 85.7%, maximum: 90.6%). These values are all fairly close to those obtained using the analysis population. Thus, the selection bias in our analysis population had little effect on our results.

## Discussion

A simple method of evaluating a patient's risk of substantial weight gain during long-term olanzapine treatment would be useful for mental health professionals. Therefore, these analyses focused on evaluating the predictive value of a single threshold for weight gain at selected early time points. We did not evaluate predictive values of baseline characteristics individually, but did evaluate their collective impact alongside our primary predictors (early weight gain and early BMI change). The importance of using early weight gain and BMI change can be seen by comparing predictions based on these values alone with predictions based on all variables taken together.

Our results suggest that evaluating patients' likelihood of substantial weight gain based on early weight gain or BMI increase is simple and effective. Using simple cut-offs for early weight gain or BMI increase in an attractive alternative to assessing individual risk of substantial weight gain by applying a complicated formula. Simple early weight gain cut-offs varied from 0.4 to 2.6 kg, depending on time point and definition of substantial weight gain. Simple early BMI increase cut-offs ranged from .13 to .83 kg/m^2^. Validation of these cut-offs in post-hoc analysis of a second patient population gave operating characteristics comparable to those found in the initial population.

Weight gain of 5 to 10 kg or more may be a potential health risk for some patients. For this reason, clinicians may find the NPV – the percentage of patients without early weight gain who remain without substantial weight gain after approximately 30 weeks – to be the most valuable tool in deciding whether patients benefiting from olanzapine therapy should remain on that therapy.

Clinicians can use the data presented in Tables 2 and 3 to predict weight gain and BMI increase based on each patient's needs and situation. For instance, these analyses found that at 3 weeks of treatment 2.0 kg was the optimal early weight cut-off for predicting a weight gain of 10 kg at endpoint (Table 2). Of the 669 patients analyzed, 265 (39.6%) had gained 2.0 kg or more at week 3. At week 30, 151 (22.6%) of the 669 patients had gained 10 kg or more. The PPV indicates approximately 39% of patients who gain 2 kg or more at week 3 will experience a weight gain of 10 kg or more by week 30. The NPV indicates approximately 88% of patients who gain less than 2 kg by week 3, will not experience a weight gain of 10 kg or more by week 30.

A simple weight gain cut off of 2 kg at 3 weeks has also been examined for bipolar disorder [16]. The observed NPV (64.5%) was less than that seen in the present study, while the PPV (64.1%) was greater.

Efficacy data at 2 weeks has also been shown to be a predictor of longer-term efficacy in olanzapine treatment [13,14]. Clinicians may be able to use patients' individual responses to acute treatment with olanzapine, both in clinical response and weight gain, as a basis for determining whether to continue olanzapine treatment, or augment with or switch to a different treatment option. In addition, if it is clinically desirable that an individual patient at potential risk of substantial weight gain continue olanzapine treatment, clinicians can proactively involve the patient in preventing or reducing further weight gain through diet, exercise, and pharmacotherapy [24-27].

We observed a correlation between improvement in PANSS score and weight gain, indicating a relationship between increased weight and improvement in symptoms. These results are consistent with previous reports of a correlation between weight gain and symptomatic improvement during treatment with antipsychotics [9,10], or placebo [28].

We also evaluated smoking status as a potential predictor of substantial weight gain. While it did not help improve prediction characteristics as such, the fact that smoking status had a moderate negative correlation with early weight change suggests that physicians may want to use slightly lower cut-offs of early weight gain when predicting substantial weight gain for smokers.

## Limitations

While the NPVs derived from these analyses are robust, the PPVs for higher levels of weight gain were modest, indicating a large degree of uncertainty associated with this phenomenon. Clinicians should continue to monitor weight gain in patients throughout treatment. Secondly, the endpoints used in this study, 28 and 30 weeks, do not represent true long-term treatment. While these endpoints are within the range where weight gain following olanzapine treatment has been reported to reach a plateau [19,20], longer-term data would be useful to confirm these results. As for any evaluation of risk factors, the results of these analyses may be affected by confounding variables not included in the predictive models. Replication of findings in larger populations and studies of longer treatment duration are needed to confirm these results. However, cut-off values derived from one study were validated using data from a second study, indicating these results are reproducible in a clinical trial population.

## Conclusion

Early weight gain during olanzapine treatment appears to be a good predictor of substantial weight gain at approximately 30 weeks. Weight gain or BMI increase cut-offs at 1, 2, 3, or 4 weeks can be used to predict potential patient risk for substantial weight gain or BMI increase during long-term treatment with olanzapine. Efficacy, potential risk of weight gain, and potential risk of other adverse events need to be considered in making the optimal treatment decision for each individual. Clinicians can determine which cut-off point and definition of substantial weight gain of BMI increase is most relevant to their patients on a case-by-case basis.

## Competing interests

All authors are employees of and minor stockholders in Eli Lilly and Company.

## Authors' contributions

IL, TAH, and VPH conceived this post-hoc study of weight gain, IL designed the statistical analysis plan and conducted the analyses. JGJ, TAH, IL and VPH contributed to planning of analyses and interpretation of results. JGJ drafted the manuscript. All authors read and approved the final manuscript.

## Pre-publication history

The pre-publication history for this paper can be accessed here:


